# Oleanolic acid cubic liquid crystal nanoparticle-based thermosensitive gel attenuates knee osteoarthritis symptoms in rats

**DOI:** 10.3389/fphar.2025.1730566

**Published:** 2025-12-19

**Authors:** Zhiqi Shi, Fan Jia, Xiaoyu Tang, Qing Li

**Affiliations:** 1 Innovation Center of Degenerative Bone & Joint Disease, Wuxi Hospital of Traditional Chinese Medicine, Wuxi, Jiangsu, China; 2 Department of pharmacy, Liangxi District of Traditional Chinese Medicine, Wuxi, Jiangsu, China; 3 Department of pharmacy, Wuxi TCM Hospital Affiliated to Nanjing University of Chinese Medicine, Wuxi, Jiangsu, China; 4 Department of TCM research, Wuxi Institute of Traditional Chinese Medicine, Wuxi, Jiangsu, China

**Keywords:** gel, knee osteoarthritis, nanoparticles, oleanolic acid, pharmacology

## Abstract

**Background:**

Knee osteoarthritis (KOA) is a prevalent degenerative joint disease with limited effective treatment options. Oleanolic acid (OA) possesses promising anti-inflammatory and cartilage-protective properties, but its clinical application is hindered by poor solubility and rapid metabolism.

**Purpose:**

This study aimed to develop an oleanolic acid-loaded liquid crystalline nanogel (OANG) for intra-articular delivery and to systematically evaluate its therapeutic effects and potential mechanisms in a rat KOA model.

**Methods:**

OA-loaded nanoparticles were prepared and incorporated into a thermosensitive Poloxamer gel base to form OANG. A papain-induced KOA rat model was established. Rats were administered OANG (high/low dose) intra-articularly, with celecoxib as a positive control. Evaluations included behavioral tests, micro-computed tomography, histological analyses (hematoxylin and eosin, transmission electron microscopy, immunohistochemistry), enzyme-linked immunosorbent assay of synovial fluid, serum, and hippocampus, Western blot (WB), network pharmacology, and molecular docking.

**Results:**

OANG exhibited sustained-release properties and improved joint lubrication. Treatment with OANG significantly alleviated KOA-induced pain and depression-like behaviors, reduced cartilage degradation and subchondral bone sclerosis, and downregulated levels of pro-inflammatory cytokines (tumor necrosis factor-α, interleukin-1β, interleukin-6) and cartilage degradation markers (C-terminal cross-linked telopeptide of type II collagen, cartilage oligomeric matrix protein) in synovial fluid. It also enhanced antioxidant capacity (increased superoxide dismutase, glutathione peroxidase; decreased malondialdehyde) and modulated the expression of key cartilage proteins (increased Collagen II; decreased matrix metalloproteinase 13; regulated glycogen synthase kinase-3β/SRY-box transcription factor 9, β-catenin, and Yes-associated protein). Furthermore, OANG ameliorated hippocampal oxidative stress and inflammation (decreased Cleaved caspase-3, Malondialdehyde; increased IL-10). Network pharmacology and docking suggested the involvement of peroxisome proliferator-activated receptor gamma, mitogen-activated protein kinase 3, prostaglandin-endoperoxide synthase 2, and pathways such as estrogen signaling and cyclic adenosine monophosphate signaling.

## Introduction

Knee osteoarthritis (KOA) is a chronic and disabling joint disorder characterized by degenerative changes in articular cartilage, synovitis, and osteophyte formation (Kloppenburg et al., 2025). Its pathogenesis is closely associated with factors such as age, obesity, trauma, and mechanical stress. With the accelerating trend of global aging, the prevalence of KOA continues to rise, rendering it a major public health concern (Liu et al., 2026). Current clinical management primarily focuses on symptomatic relief and functional improvement, commonly involving the use of nonsteroidal anti-inflammatory drugs (NSAIDs), intra-articular corticosteroid injections, or hyaluronic acid supplementation (Liu et al., 2025). However, these approaches generally offer only short-term alleviation without delaying disease progression (Zhang et al., 2025a). Long-term application is also associated with significant adverse effects, including gastrointestinal and cardiovascular complications, as well as potential cartilage damage, highlighting the dual challenges of limited efficacy and safety (Xu et al., 2025).

Oleanolic acid (OA), a naturally occurring pentacyclic triterpenoid found in medicinal plants such as *Ligustrum lucidum* and *Hedyotis diffusa*, has garnered increasing interest due to its notable anti-inflammatory, antioxidant, and cartilage-protective properties (Liu et al., 2025). Studies have shown that OA counteracts KOA through multiple mechanisms: it suppresses key inflammatory signaling pathways such as nuclear factor-kappa B (NF-κB) and mitogen-activated protein (MAPK) kinases, downregulates the expression of cyclooxygenase-2 (COX-2) and inducible nitric oxide synthase (iNOS), and reduces the production of pro-inflammatory cytokines such as interleukin-1β (IL-1β) and tumor necrosis factor-α (TNF-α) (Han et al., 2025; Lee et al., 2013; Melo et al., 2016). Moreover, OA mitigates oxidative stress and inhibits extracellular matrix degradation in chondrocytes, thereby slowing cartilage destruction (Turgut et al., 2025). These multifaceted pharmacological properties position OA as a promising therapeutic candidate for KOA.

Despite its potential, the clinical translation of OA is hindered by its unfavorable physicochemical and pharmacokinetic properties (Funayama et al., 2025). Specifically, OA exhibits very low aqueous solubility, leading to poor oral bioavailability and difficulty in maintaining effective blood concentrations (Ma et al., 2025). It also undergoes rapid metabolism *in vivo*, resulting in a short half-life that necessitates frequent dosing, thereby increasing the risk of side effects and reducing patient compliance. Furthermore, achieving targeted and sustained delivery to articular tissues remains a major challenge in OA-based therapy for KOA (Tu et al., 2025).

Phase-change gels present a promising strategy for KOA treatment, leveraging their intelligent thermo-responsive behavior (Qiu et al., 2021). These systems are injectable as a liquid *in vitro* and undergo transition to a semi-solid gel at body temperature, facilitating prolonged local retention within the joint (Qian et al., 2025). During movement, the gel can revert to a more fluid state, providing additional lubrication (Guo et al., 2025). Serving as a localized “drug depot,” such a system enables sustained release, enhanced targeting, prolonged efficacy, and improved patient compliance.

To overcome the delivery limitations of OA, our previous research developed an OA cubic liquid crystal nanoparticle-based thermosensitive gel (OANG) for intra-articular administration (Shi et al., 2021a; Shi et al., 2025a). By integrating nanotechnology to enhance solubility and a gel matrix for localized retention and controlled release, this system is designed to prolong the intra-articular residence time and bioavailability of OA. In the present study, the therapeutic potential and underlying mechanisms of OA-Nanogel—particularly its anti-inflammatory and cartilage-protective effects—will be systematically evaluated in an experimental KOA model. The findings are expected to provide an experimental foundation and theoretical support for the development of novel targeted OA delivery systems and treatment strategies for KOA.

## Materials and methods

OA was purchased from Shanghai Yuanyet Biotechnology Co., Ltd. (Shanghai, China). Clomipramine hydrochloride tablets were provided by Xuzhou Enhua Pharmaceutical Group Co., Ltd. (Xuzhou, China). Polyethylene glycol (PEG)-2000, Poloxamer F127, and F68 were sourced from Sigma-Aldrich Co., Ltd. (Shanghai, China). Sodium dodecyl sulfate (SDS) and phytantriol were obtained from Shanghai Aladdin Biochemical Technology Co., Ltd. (Shanghai, China). Isoflurane was supplied by Lunambet Pharmaceutical Co., Ltd. (Linyi, China).

Commercial enzyme-linked immunosorbent assay (ELISA) kits for 5-hydroxytryptamine (5-HT), interleukin (IL)-1β, IL-6, IL-17, IL-18, C-terminal cross-linked telopeptide of type II collagen (CTX-II), Transforming growth factor beta (TGF-β) cartilage oligomeric matrix protein (COMP), TNF-α, superoxide dismutase (SOD), catalase (CAT), glutathione peroxidase (GSH-Px), malondialdehyde (MDA), and brain-derived neurotrophic factor (BDNF) were acquired from Jiangsu Keygen BioTECH Co., Ltd. (Nanjing, China).

The following antibodies were used: mouse anti-β-actin (Lot: KGC6101-1; MW: 43 kDa; dilution: 1:1,000) from Jiangsu Keygen BioTECH Co., Ltd. (Nanjing, China); rabbit anti-Collagen II (Lot: 27418-1-AP; MW: 56 kDa; dilution: 1:2,000), rabbit anti-SOX9 (Lot: 27528-1-AP; MW: 56 kDa; dilution: 1:2,000), rabbit anti-matrix metalloproteinase 13 (MMP13) (Lot: 24153-1-AP; MW: 54 kDa; dilution: 1:2,000), rabbit anti-glycogen synthase kinase 3β (GSK3β) (Lot: 18252-1-AP; MW: 47 kDa; dilution: 1:1,000), rabbit anti-β-catenin (Lot: 51067-2-AP; MW: 78–80 kDa; dilution: 1:2,000), and rabbit anti-Yes-associated protein (YAP) (Lot: 13584-1-AP; MW: 55 kDa; dilution: 1:200) from Wuhan Sanying Biotechnology Co., Ltd. The MaxVision Kit (Rabbit) (Lot: KIT-5005) was purchased from Fuzhou Maixin Biotechnology Co., Ltd. (Fuzhou, China).

All materials were used as received without further purification.

### Preparation of oleanolic acid-loaded cubosomal nanoparticles

Oleanolic acid-loaded liquid crystalline nanoparticles were prepared according to our previously established precursor method (Shi et al., 2021b). Briefly, phytantriol (1.6 g) and OA (0.8 g) were dissolved in absolute ethanol (2.5 mL) and sonicated at 80% power for 30 min. The resulting organic phase was added dropwise into an aqueous phase (20 mL) containing the stabilizer Poloxamer F127 under continuous agitation to form a crude dispersion. This dispersion was further sonicated in pulsed mode (0.5 s pulse, 0.5 s pause) at 40% maximum power for 10 min to yield a milky, homogeneous nanoparticle suspension with reduced particle size.

### Preparation of phase-change gels

The phase-change gel was fabricated based on our earlier formulation screening work (Shi et al., 2025b). The as-prepared OA-LCNP dispersion (1.5 g) was accurately weighed into a vial. Poloxamer F68 (0.1 g) and F127 (0.4 g) were then evenly dispersed onto the nanoparticle solution, and the mixture was refrigerated at 4 °C overnight to allow complete swelling and dissolution of the Poloxamers, forming a uniform gel precursor. Finally, PEG-2000 was incorporated at 1% (w/w) of the total gel mass.

Consistent with our previous characterization data, the resulting OANG exhibits thermosensitive sustained-release behavior, gelling at 34 °C and releasing the drug in accordance with the Higuchi kinetic model. Its lubrication properties—characterized by low fluidity and a moderate friction coefficient at body temperature—promote adhesion to the articular cartilage surface and help reduce wear.

### Transmission electron microscopy (TEM) photographing of gel

The samples were observed directly using an F200 field-emission transmission electron microscope (JEOL Ltd., Tokyo, Japan). A carrier grid was held with tweezers and immersed in the solution to collect the droplets. After the droplets on the supporting film were fully dried, the samples were observed under the electron microscope. The nanoparticle morphology was examined at an accelerating voltage of 120 kV (Vedadghavami et al., 2020).

### Network pharmacology analysis of OA against knee osteoarthritis

Potential therapeutic targets of OA were retrieved from the Swiss Target Prediction database (Zhang et al., 2021). Disease-related targets associated with KOA were collected from the OMIM, Drug Bank, and Gene Cards databases. The intersection of these target sets was identified using Venny 2.1. The overlapping targets were imported into the STRING database to construct a protein–protein interaction (PPI) network, with the species restricted to *Homo sapiens* and a confidence threshold set at > 0.9. Isolated nodes were removed, and the resulting network was visualized and analyzed using Cytoscape 3.7.1 (Que et al., 2022).

The common targets were subsequently submitted to the DAVID database for Kyoto Encyclopedia of Genes and Genomes (KEGG) pathway enrichment analysis, using “OFFICIAL_GENE_SYMBOL” as the identifier and limiting the analysis to *Homo sapiens*. A “Component–Target–Pathway” network was constructed with Cytoscape to illustrate the multi-scale interactions (Wang et al., 2025a).

### Animals

#### Animals and experimental design

Fifty Sprague-Dawley (SD) rats were randomly assigned to five groups (n = 10 per group): normal control (Control), model (Model), high-dose OANG (OANG-H, 50 mg/kg), low-dose OANG (OANG-L, 25 mg/kg), and positive control (CX, celecoxib 20 mg/kg).

To establish the KOA model, rats were anesthetized with isoflurane at an inhalation flow rate of 0.5–0.7 L/min, and then the modeling groups received five intra-articular injections of a mixture containing 3% papain and 0.03 mol/L L-cysteine (0.2 mL per injection) into the left knee cavity at 3-day intervals (Yang et al., 2021). The Control group was injected with an equal volume of normal saline. All animals were housed under identical conditions throughout the experiment. Successful modeling was confirmed through behavioral, imaging, and histological assessments.

### Drug administration

Before each intra-articular injection administration, isoflurane anesthesia is used with an inhalation flow rate of 0.5–0.7 L/min. After modeling, the Control and Model groups received an intra-articular injection of normal saline (0.2 mL). The OANG-H and OANG-L groups were administered 0.2 g of the corresponding gel formulation, while the CX group received celecoxib suspension via oral gavage (20 mg/kg) (Shi et al., 2025a).

### Sample collection and processing

Two weeks post-treatment, rats were anesthetized by intraperitoneal injection of 3% sodium pentobarbital (40 mg/kg body weight). Synovial fluid was aspirated from the knee joint using a 1 mL syringe, centrifuged at 2000 r min^-1^ for 10 min, and the supernatant stored at −80 °C. Blood was collected from the abdominal aorta and centrifuged at 3,000 r min^-1^ for 15 min; serum was aliquoted and stored at −80 °C (Mei et al., 2021).

After blood collection, the abdominal aorta was incised and kept open under anesthesia to allow exsanguination. Vital signs were monitored until respiration and heartbeat ceased (approximately 1–3 min). Death was confirmed by absence of corneal reflex and limb movement. The brain was rapidly excised on ice and fixed in paraformaldehyde (Rai and Pham, 2018).

The left knee joint was dissected, and the intact joint along with adjacent femur and tibia was immersed in paraformaldehyde for fixation. Cartilage samples were harvested for pathological sectioning. The right knee joint was similarly processed, and cartilage tissue blocks were carefully excised, fixed in 5% glutaraldehyde, and stored at −80 °C. An additional thin cartilage specimen was snap-frozen in liquid nitrogen. During sampling, surrounding muscle and tendon tissues were thoroughly removed (Ceccarelli et al., 2017).

### Sucrose preference test

All animals were acclimatized to consuming 1% sucrose solution prior to testing. After 12 h of fasting, rats were given access to 1% sucrose in place of drinking water for 1 h as training. The formal test began after an additional 2-h fast. During testing, two visually identical bottles—one containing purified water and the other 1% sucrose—were placed in each cage. Bottle weights were recorded before and after the 1-h test period, and sucrose consumption was calculated accordingly (Zhou et al., 2022).

### Micro-CT examination and analysis of rat knee joints

Knee joints from each group were scanned using a NEMO-Micro CT system (NMC200, Pingsheng Medical Technology Co., Ltd., Suzhou, China) with the following parameters: Cruiser 2.0 software, 8.5 μm resolution, 60 kV voltage, and 500 μA current. The region of interest (ROI) was reconstructed and analyzed using Avatar Research Workplace 3.0 software. Evaluated parameters included subchondral bone mineral density (BMD), bone volume (BV), and bone surface area (BS) (Lin et al., 2026).

### Observation of chondrocyte ultrastructure via TEM

Cartilage tissues (approximately 1 mm^3^) were immediately fixed in 2.5% glutaraldehyde, followed by phosphate-buffered saline (PBS) rinsing and post-fixation in 1% osmium tetroxide. Samples were dehydrated through a graded ethanol and propylene oxide series, embedded in epoxy resin, and polymerized in an oven. Ultrathin sections (70 nm) were cut, collected on copper grids, stained with lead citrate, and observed under a transmission electron microscope operating at 80 kV (Lu et al., 2025).

### Observation of articular cartilage morphology by HE staining

Fixed knee joint specimens underwent dehydration, clearing, paraffin infiltration, embedding, and sectioning. Paraffin sections were dewaxed, stained with HE, dehydrated, and mounted. Morphological changes in articular cartilage were observed and imaged under a light microscope for analysis (Sun et al., 2025).

### ELISA

Levels of cross-linked CTX-II, COMP, TNF-α, IL-1β, SOD, CAT, GSH-Px, and MDA in synovial fluid were measured using commercial ELISA kits, according to the manufacturers’ protocols. Similarly, IL-18, IL-17, IL-6, IL-1β, and TNF-α levels in serum, as well as IL-1β, IL-10, SOD, MDA, and BDNF levels in hippocampal tissue, were also quantified by ELISA (Shi et al., 2025b).

Briefly, all kits were equilibrated to room temperature before use. Samples and standards were added to the appropriate wells, followed by the addition of enzyme conjugate. After sealing and incubation, the plates were washed five times with diluted wash buffer. Chromogen and stop solutions were then applied, and the optical density (OD) of each well was measured at 450 nm. Sample concentrations were determined based on standard curve regression.

### Immunohistochemical (IHC) staining

For immunohistochemical detection of β-catenin and YAP, fixed specimens were dehydrated, transparentized, embedded in paraffin, and sectioned. After deparaffinization, antigen retrieval was performed using citrate-based buffer (pH 6.0) in a microwave heater. Sections were then incubated with 3% hydrogen peroxide in the dark for 25 min to block endogenous peroxidase activity, followed by blocking with 3% bovine serum albumin (BSA) for 30 min. Primary antibodies (1:100 dilution) were applied and incubated overnight at 4 °C. After washing, secondary antibodies (1:1,000 dilution) were added and incubated for 50 min at room temperature. Color development was carried out using diaminobenzidine (DAB), and sections were counterstained with hematoxylin, dehydrated, and mounted. Staining results were observed under a microscope and analyzed using ImageJ software (Wang et al., 2025b).

### Immunofluorescence staining

Paraffin-embedded sections were deparaffinized in xylene and rehydrated through a graded ethanol series, followed by PBS rinses. Antigen retrieval was conducted using citrate buffer under microwave irradiation (medium-high power, 30 min). After cooling and PBS washes, endogenous peroxidase activity was quenched with 3% H_2_O_2_ in methanol (10 min, room temperature (RT)). Non-specific binding was blocked with goat serum (20 min, RT). Sections were incubated overnight at 4 °C with primary antibodies (diluted 1:400), washed with PBS, and then incubated with fluorescein isothiocyanate (FITC)-conjugated secondary antibodies at 37 °C for 1 h in the dark. Nuclei were counterstained with 4′,6-diamidino-2-phenylindole (DAPI) for 5 min at RT. Slides were mounted with anti-fade medium and scanned using an Olympus VS200 slide scanner (Olympus, Tokyo, Japan). Tissue-specific optimization was applied for antigen preservation in neural and cardiac samples. Cleaved caspase-3 expression was quantified using ImageJ software (version 1.54d) (Lu et al., 2025).

### Western blot analysis

Cartilage tissues were ground into powder in liquid nitrogen-precooled mortars. Approximately 20 mg of powder was lysed in 200 μL radioimmunoprecipitation assay (RIPA) buffer containing phenylmethylsulfonyl fluoride (PMSF) (99:1), vortexed, and centrifuged at 12,000 rpm for 10 min at 4 °C. The supernatant was collected, and protein concentration was determined using a bicinchoninic acid (BCA) assay kit. Proteins were denatured with 5× loading buffer, separated by tricine–sodium dodecyl sulfate–polyacrylamide gel electrophoresis (Tricine-SDS-PAGE) at 120 V for 50 min, and transferred onto 0.22 μm polyvinylidene fluoride (PVDF) membranes at 110 V for 90 min. Membranes were blocked, incubated with primary antibodies (1:1,000) at 4 °C overnight, and then with corresponding secondary antibodies (1:3,000). Protein bands were visualized using enhanced chemiluminescence (ECL) substrate. β-actin or glyceraldehyde-3-phosphate dehydrogenase (GAPDH) was used as the loading control. Band intensities were quantified using ImageJ 1.5.4 (Lu et al., 2025).

### Statistical analyses

The sample size for each group (n = 10) was determined by *a priori* power analysis using G*Power software (version 3.1.9.7). Based on our pilot experiments and literatures (Abdel-Aziz et al., 2021; Tschon et al., 2026), we anticipated a large effect size (f = 0.8) for the primary outcome measure (e.g., mechanical allodynia threshold or cartilage degradation score). With an alpha (α) level of 0.05 and a desired power (1-β) of 90% for a one-way ANOVA, the analysis indicated that a minimum of 8 animals per group would be required. To account for potential attrition during the experimental period, we increased the sample size to 10 rats per group (Kauppinen et al., 2025; Lee et al., 2024).

All statistical analyses were performed using SPSS 21.0 software (IBM Corporation, United States) and all data were presented as means ± standard error of the mean (SEM). Statistical significance between various treatment groups was evaluated using one-way analysis of variance (ANOVA) with the Bonferroni *post hoc* test. Statistical significance was defined as a two-tailed P-value <0.05 (*, # indicate p < 0.05; **, ## indicate p < 0.01; NS indicates not significant) (Tu et al., 2025).

## Results

### TEM photographing

**FIGURE 1:**
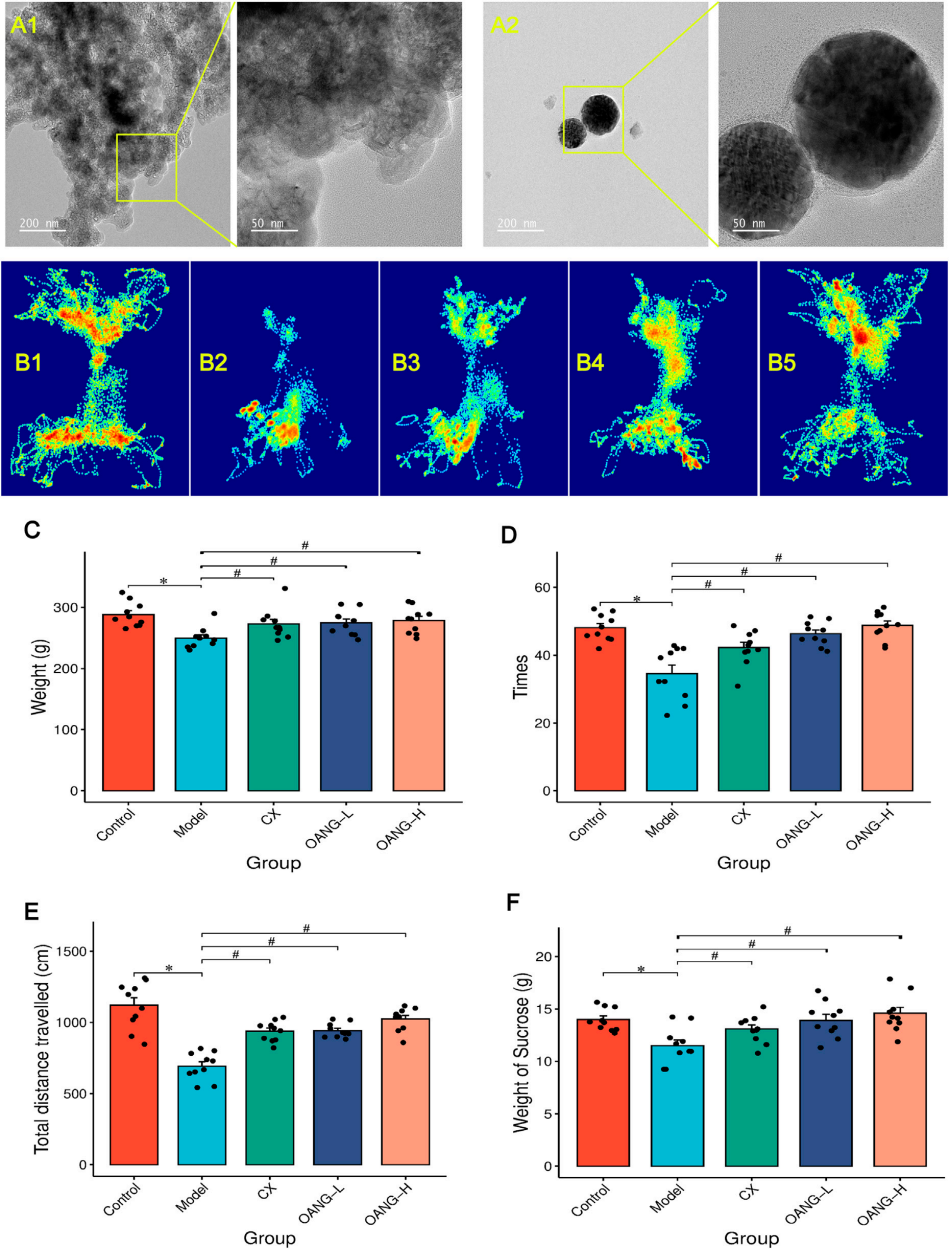
Results of the behavior test in rats (n = 10/group). **(A)** TEM results (A1: OA suspension in gel; A2: OANG); **(B)** Shuttle track of rats (1: Control, 2: Model, 3: CX, 4: OANG-L, and 5: OANG-H groups); **(C)** Body weight of rats; **(D)** Analysis of the box shuttle test results; **(E)** Total distance travelled test; **(F)** Results of sucrose preference tests. Comparison with the control group, *P < 0.05; compared with the model group, #P < 0.05.

TEM analysis revealed that direct addition of OA to the gel resulted in highly uneven distribution (Figure 1). The gel matrix without OA appeared nearly transparent, while areas containing OA showed significant aggregation, suggesting inadequate dispersion (Liu et al., 2026). In contrast, TEM images of OA nanoparticles incorporated in a thermosensitive Poloxamer gel matrix demonstrated nearly homogeneous dispersion within the gel, which is consistent with our previous findings (Shi et al., 2025a).

### Behavioral analysis

As illustrated in Figure 1B, the movement patterns and dwelling time at different locations varied among groups. Control rats, driven by curiosity, frequently shuttled through the central aperture and explored the entire arena (Figure 1B1). In contrast, Model group animals showed markedly reduced shuttling frequency and locomotor activity, likely due to KOA-related pain and diminished exploratory behavior (Figure 1B2). Both the CX and OANG treatment groups exhibited improved mobility, with increased travel distance and shuttling times compared to the Model group (Figures 1B3–B5).

Body weight was significantly lower in the Model group than in the Control group (P < 0.05, Figure 1C). Both OANG-high dose (OANG-H) and OANG-low dose (OANG-L) groups, as well as the CX group, showed significant weight recovery relative to the Model group (P < 0.05).

The number of shuttling events was significantly reduced in the Model group (P < 0.05, Figure 1D), but was markedly increased in the OANG-H, OANG-L, and CX groups (P < 0.05). Similarly, total distance travelled was significantly lower in the Model group (P < 0.05, Figure 1E), and all treatment groups showed significant recovery (P < 0.05).

In the sucrose preference test, sucrose consumption was significantly lower in the Model group (P < 0.05, Figure 1F). Both OANG doses and the CX group led to a significant increase in sucrose intake (P < 0.05).

### OANG alleviates articular cartilage wear and extracellular matrix damage

**FIGURE 2:**
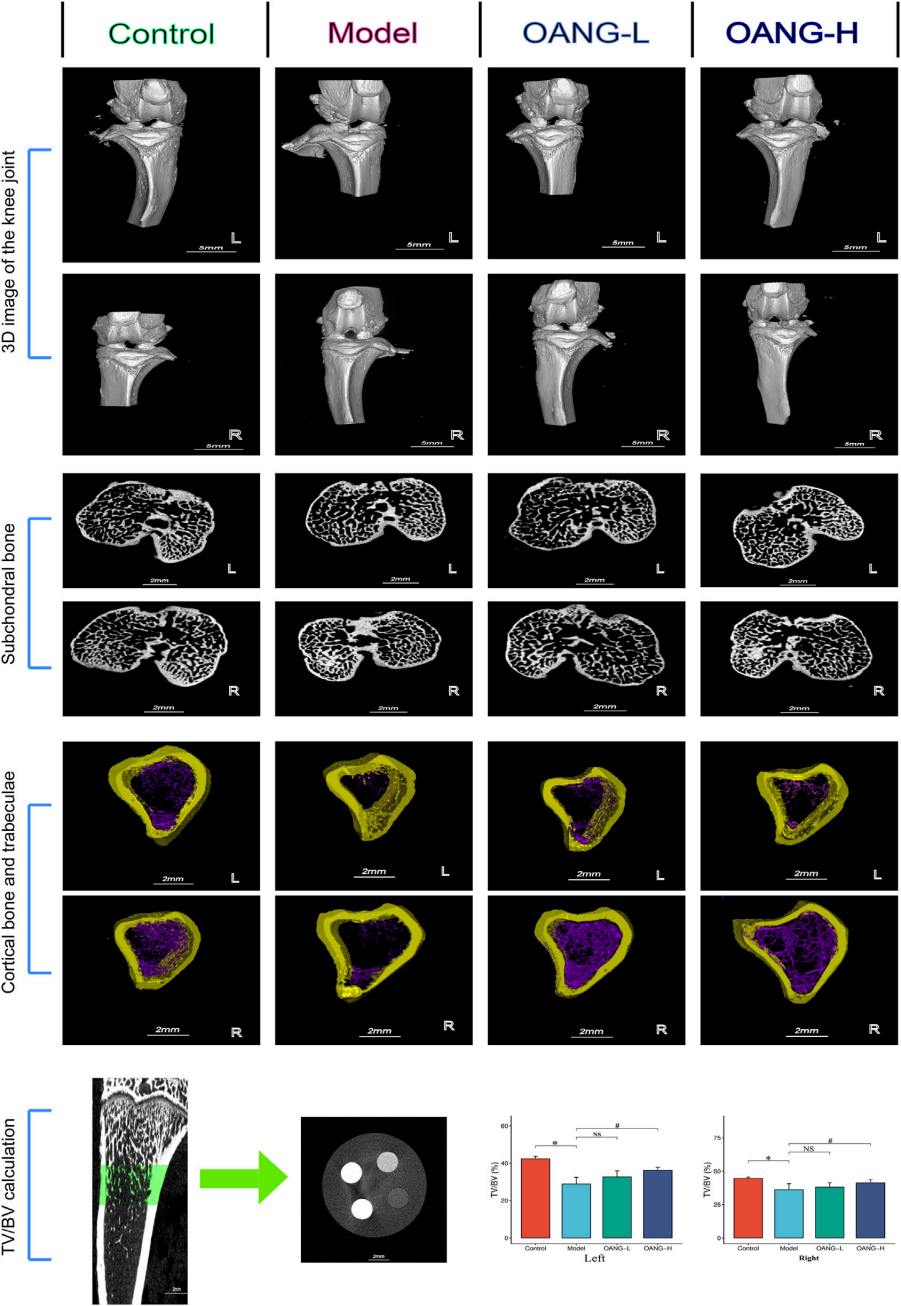
Rat imaging findings results. Comparison with the control group, *P < 0.05; compared with the model group, #P < 0.05.

**FIGURE 3:**
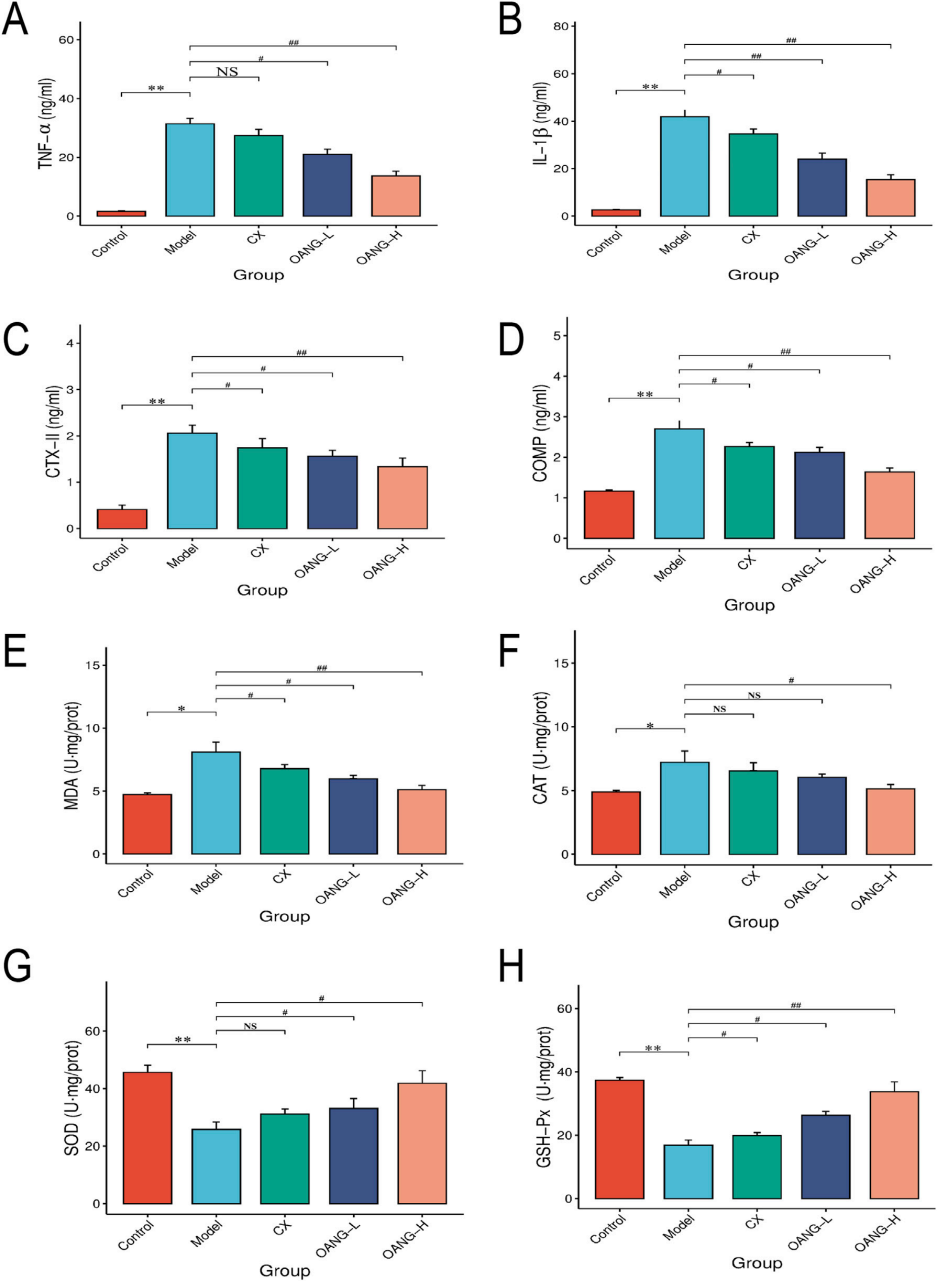
Synovial fluid inflammatory factor test results. Comparison with the control group, *P < 0.05, ** P < 0.01; compared with the model group, #P < 0.05, ##P < 0.01.

Micro-CT 3D reconstruction of the knee joint revealed decreased subchondral bone porosity in the Model group, indicative of osteosclerosis. OANG treatment reduced cartilage damage and increased porosity (Figures 2, 3D structure). The joint space was larger in the modeled left knee than in the right knee, but after OANG administration, porosity between the two knees became comparable, suggesting improved joint integrity.

Cross-sectional analysis showed subchondral trabecular sclerosis in the Model group, likely resulting from mechanical wear and compensatory bone remodeling (Figure 2, subchondral bone). Sclerosis was more severe in the modeled left knee, but OANG treatment restored trabecular structure similarity between knees.

Avatar software-based analysis indicated trabecular reduction and loss in the Model group, which was ameliorated by OANG (Figure 2, cortical bone and trabeculae). Trabecular bone volume fraction was significantly reduced in the Model group; it was significantly improved in the OANG-H group but not in the OANG-L group (Figure 2, TV/BV calculation).

### Effects on inflammatory factors and antioxidant indicators in synovial fluid

Levels of TNF-α, IL-1β, CTX-II, COMP, MDA, and CAT were significantly elevated in the Model group (P < 0.05, Figures 3A–F). OANG-H and OANG-L significantly reduced these levels (P < 0.05). The CX group also showed significant reductions in IL-1β, CTX-II, COMP, and MDA (P < 0.05), but not significant in TNF-α or CAT (P > 0.05).

In contrast, GSH-Px and SOD activities were significantly lower in the Model group (P < 0.01, Figures 3G,H). Both OANG doses significantly increased these activities (P < 0.05). The CX group showed significantly increased GSH-Px (P < 0.05) but not SOD (P > 0.05).

### Effects on the morphology of rat articular cartilage tissue

**FIGURE 4:**
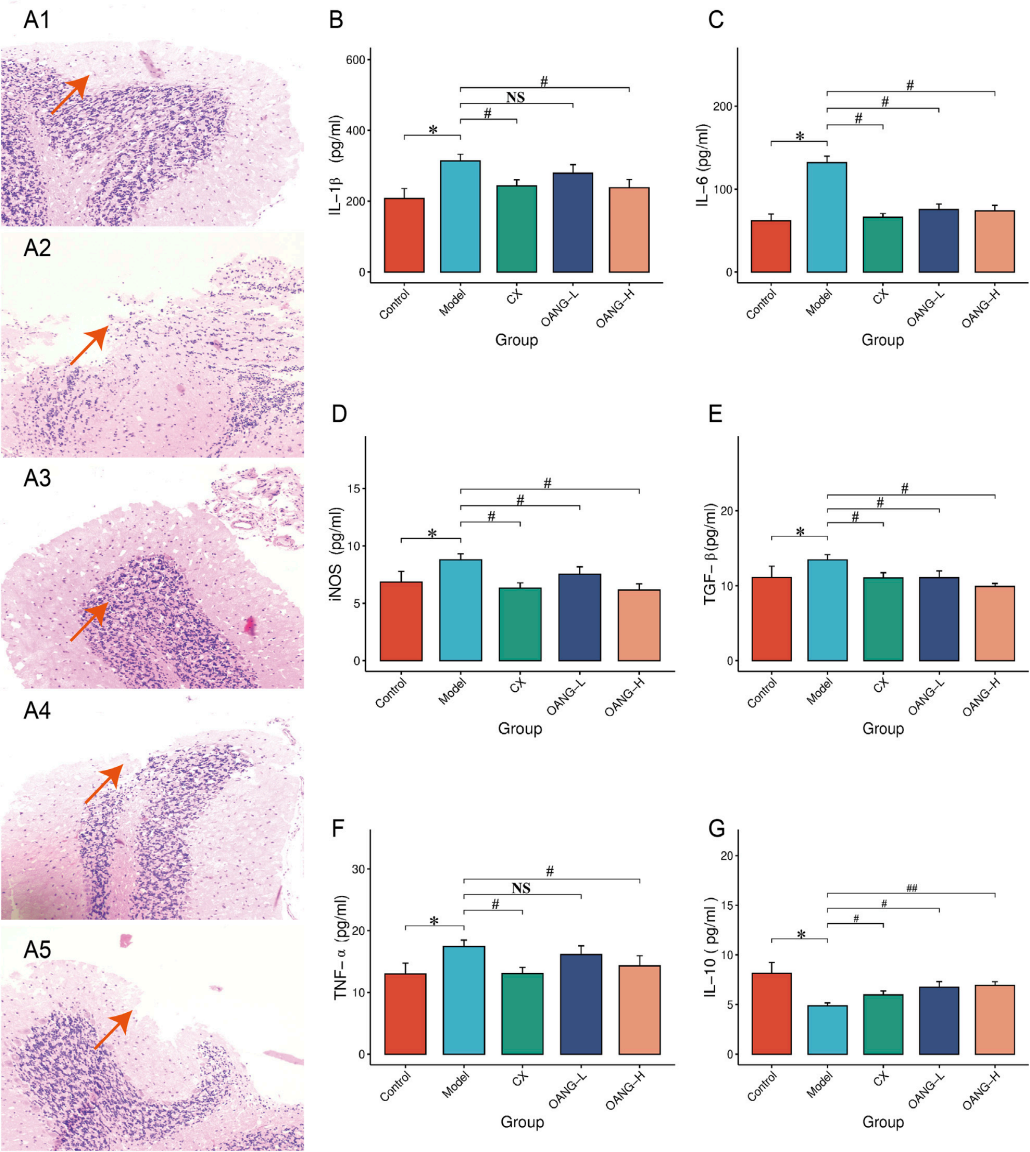
Cartilage histopathology and blood inflammatory factor test results. **(A)** histopathology (1: Control, 2: Model, 3: CX, 4: OANG-L, and 5: OANG-H groups; ‘100 ); **(B–G)** levels of inflammatory factors. Comparison with the control group, *P < 0.05; compared with the model group, #P < 0.05, ##P < 0.01.

In the Control group, chondrocytes were evenly distributed, the articular surface was smooth, and layers were distinct (Figure 4A1). The Model group exhibited cartilage defects, disorganized cell arrangement, unclear layering, a blurred tidemark, and vascular invasion (Figure 4A2). All treatment groups showed improved cartilage morphology, with increased cell number, more regular arrangement, and minimal vascular invasion, though the tidemark remained somewhat indistinct (Figures 4A3–A5).

### Effect on inflammatory factor expression in articular cartilage

Levels of IL-1β, IL-6, iNOS, TGF-β, and TNF-α were significantly elevated in the Model group (P < 0.05, Figures 4B–F). OANG-H significantly reduced all these markers (P < 0.05). OANG-L significantly decreased IL-6, iNOS, and TGF-β (P < 0.05), but not IL-1β or TNF-α. The CX group also showed significant reductions in all five markers (P < 0.05). IL-10 was significantly decreased in the Model group (P < 0.05, Figure 4G), and all treatment groups significantly increased its level, with OANG-H showing the most pronounced effect (P < 0.01).

### Network pharmacology analysis

**FIGURE 5:**
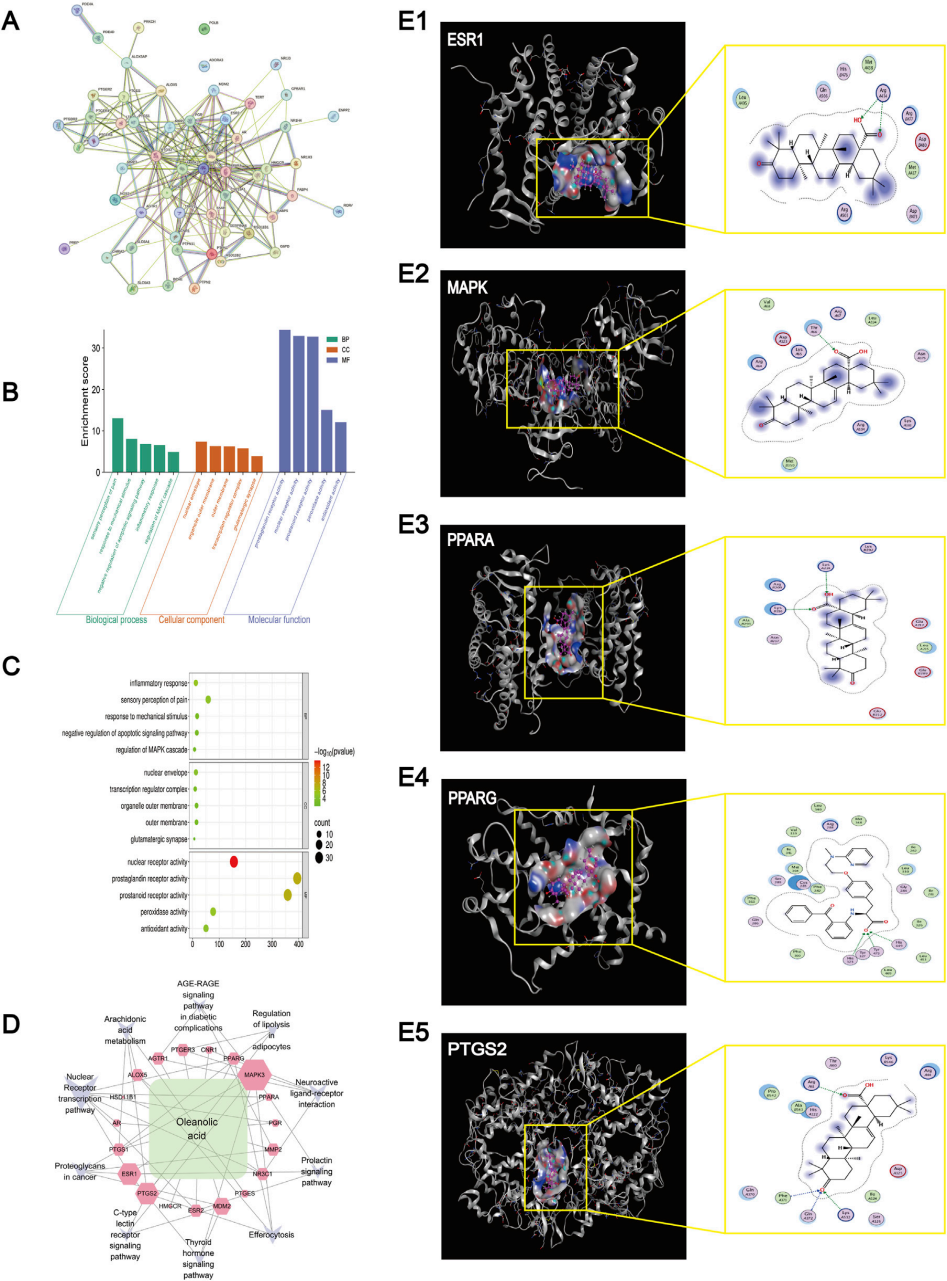
Network pharmacology analysis and Molecular docking results. **(A)** Results of the PPI network analysis of genes related to Oleanolic Acid treatment for osteoarthritis. **(B)** KEGG enrichment results of genes and pathways associated with Oleanolic Acid treatment for osteoarthritis. **(C)** Bubble chart of GO (Gene Ontology) analysis for genes and pathways related to Oleanolic Acid treatment for osteoarthritis (The larger the bubble, the higher the degree of relevance/association). **(D)** Network diagram of Oleanolic Acid, gene targets, and pathways (The larger the label, the higher the degree of relevance/association). **(E)** Molecular docking results for ESR1 (protein 9BQE), MAPK (protein 2ZOQ), PPARA (protein 3ET1), PPARG (protein 9CK0) and PTGS2 (protein 5F19).

Using Swiss Target Prediction, 100 potential targets of OA were identified. A total of 6,521 knee osteoarthritis (KOA)-related targets were retrieved from OMIM, Drug Bank, and Gene Cards. Venn analysis using Venny 2.1 revealed 56 overlapping targets. After excluding two targets without interactions (POLB and ADORA3), PPI network analysis yielded 272 nodes and 2,204 interaction pairs, with an average degree of 8.03, betweenness centrality (BC) of 0.0239, neighborhood connectivity (NC) of 12.40, and closeness centrality of 0.4535, indicating multi-target complexity in KOA treatment (Figure 5A).

Gene Ontology (GO) analysis of the 24 key targets from PPI results identified 783 entries; after filtering (p < 0.05), 208 significant terms remained (217 biological process [BP], 5 cellular component [CC], 31 molecular function [MF]) (Figures 5B,C). Top BP terms included inflammatory response regulation, cellular response to hormone stimulus, and nuclear receptor signaling pathways. Key CC terms involved the nuclear envelope and transcription regulator complex. Main MF terms included nuclear receptor activity and steroid binding.

KEGG analysis identified 10 osteoarthritis-related pathways. A “Component–Target–Pathway” network constructed in Cytoscape3.7.4 highlighted key targets such as peroxisome proliferator-activated receptor gamma (PPARG), prostaglandin-endoperoxide synthase 2 (PTGS2), mitogen-activated protein kinase 3 (MAPK3), PPAR alpha (PPARA), and estrogen receptor 1 (ESR1) (Figure 5D). These were enriched in pathways including estrogen signaling, arachidonic acid metabolism, serotonergic synapse, and cyclic adenosine monophosphate (cAMP) signaling.

### Molecular docking results

Molecular docking analysis was performed to evaluate the binding interactions between oleanolic acid-derived ligands (O1, O2, O3, O2 17) and five key therapeutic targets: ESR (PDB: 9BQE), MAPK (PDB: 2ZOQ), PPARA (PDB: 3ET1), PPARG (PDB: 9CK0), and PTGS2 (PDB: 5F19). The results indicated stable binding interactions, characterized by favorable binding energies and specific hydrogen-bond contacts (Figures 5E1–E5; Table 1).

**TABLE 1:** Molecular docking results.

Protein (PDB ID)	Ligand	Receptor residue	Interaction	Distance (Å)	E (kcal/mol)
9BQE (ESR)	O 2	ARG 434 (A) NH2	H-acceptor	3.36	−0.9
	O 3	ARG 434 (A) NH1	H-acceptor	3.11	−5.3
2ZOQ (MAPK)	O 3	THR 66 (A) OG1	H-acceptor	2.94	−2.0
3ET1(PPARA)	O 2	LYS 224 (A) NZ	H-acceptor	3.15	−2.4
	O 3	LYS 216 (B) CE	H-acceptor	3.28	−0.6
	O 3	LYS 216 (B) NZ	H-acceptor	3.18	−6.2
9CK0(PPARG)	O2 17	HIS 323 (A) NE2	H-acceptor	2.93	−4.4
	O2 17	HIS 449 (A) NE2	H-acceptor	2.96	−3.8
	O2 17	TYR 473 (A) OH	H-acceptor	2.49	−1.4
5F19(PTGS2)	O 1	PHE 371 (A) CA	H-acceptor	3.52	−0.6
	O 1	GLN 372 (A) N	H-acceptor	2.94	−1.4
	O 1	LYS 532 (A) NZ	H-acceptor	3.68	−3.1
	O 3	ARG 61 (A) CD	H-acceptor	3.07	−0.7

Key interactions were primarily mediated by hydrogen bonding. Specific ligand–residue interactions included: For ESR (9BQE): O2 formed a hydrogen bond with ARG 434(A) NH2 (3.36 Å, −0.9 kcal/mol), and O3 bonded with ARG 434(A) NH1 (3.11 Å, −5.3 kcal/mol). For MAPK (2ZOQ): O3 interacted with THR 66(A) OG1 (2.94 Å, −2.0 kcal/mol). For PPARA (3ET1): O2 bound to LYS 224(A) NZ (3.15 Å, −2.4 kcal/mol), while O3 formed bonds with LYS 216(B) CE (3.28 Å, −0.6 kcal/mol) and LYS 216(B) NZ (3.18 Å, −6.2 kcal/mol). For PPARG (9CK0): O2 17 established multiple contacts with HIS 323(A) NE2 (2.93 Å, −4.4 kcal/mol), HIS 449(A) NE2 (2.96 Å, −3.8 kcal/mol), and TYR 473(A) OH (2.49 Å, −1.4 kcal/mol). For PTGS2 (5F19): O1 interacted with PHE 371(A) CA (3.52 Å, −0.6 kcal/mol), GLN 372(A) N (2.94 Å, −1.4 kcal/mol), and LYS 532(A) NZ (3.68 Å, −3.1 kcal/mol), while O3 bound to ARG 61(A) CD (3.07 Å, −0.7 kcal/mol). Notably, PPARA (3ET1) exhibited the strongest binding affinity, with O3 showing a binding energy of −6.2 kcal/mol toward LYS 216(B) NZ. PPARG (9CK0) engaged in multiple stable interactions via O2 17, and the ligand O3 demonstrated broad target binding capability across ESR, PPARA, and PTGS2.

### Effects on the ultrastructure of chondrocytes in KOA rats

**FIGURE 6:**
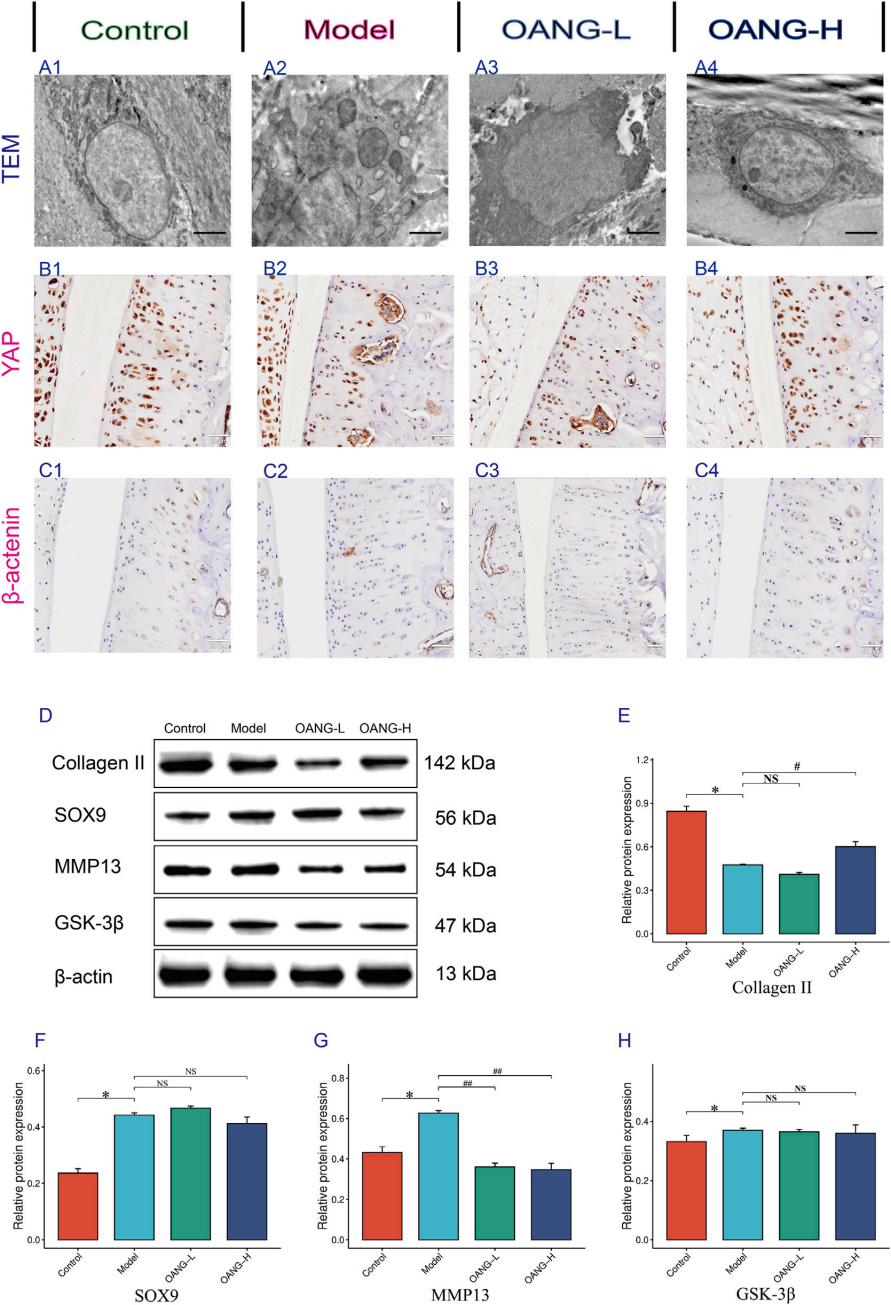
Results of the behavior test in rats (n = 10/group). **(A–C)** IHC results of Articular Cartilage. **(D–H)** WB results of articular Cartilage. Comparison with the control group, *P < 0.05; compared with the model group, #P < 0.05.

TEM revealed intact chondrocyte structure, normal organelle morphology, and uniform extracellular matrix distribution in the control group (Figure 6A1). In contrast, the Model group exhibited severe ultrastructural damage, including organelle swelling and disrupted matrix organization (Figure 6A2). The OANG-low dose (OANG-L) group showed partial improvement in chondrocyte morphology and matrix integrity, though not to the level of the Control group (Figure 6A3). The OANG-high dose (OANG-H) group displayed near-normal ultrastructure, with well-preserved organelles and homogeneous matrix distribution, indicating a dose-dependent restorative effect of OANG (Figure 6A4).

### IHC staining of YAP and β-catenin

IHC analysis showed uniform positive expression of YAP in the cartilage of Control animals, which was disrupted in the Model group (Figure 6B). OANG-L partially restored YAP expression, while OANG-H resulted in a distribution similar to the Control group, suggesting dose-dependent recovery. Similarly, β-catenin expression was uniform in Controls and aberrant in the Model group (Figure 6C). Both OANG treatments promoted recovery, with OANG-H showing expression patterns closest to the Control group.

### Western blot (WB) analysis of cartilage proteins

WB results indicated that collagen II expression was significantly downregulated in the Model group compared to Controls (p < 0.05). OANG-L slightly increased its expression, though not significantly, while OANG-H led to a significant upregulation (p < 0.05), nearly restoring it to Control levels (Figures 6D,E). SRY-box transcription factor 9 (SOX9) expression was significantly elevated in the Model group (p < 0.05). Although neither OANG dose caused significant changes, OANG-H showed a trend toward normalization (Figure 6F). MMP13 was significantly increased in the Model group (p < 0.05), and both OANG-L and OANG-H significantly suppressed its expression (p < 0.05) (Figure 6G). Glycogen synthase kinase-3β (GSK-3β) expression was also higher in the Model group (p < 0.05). While neither OANG dose induced significant changes, OANG-H again showed a trend toward Control levels, suggesting a moderate regulatory effect (Figure 6H).

### Effects on protein expression in hippocampal tissue

**FIGURE 7:**
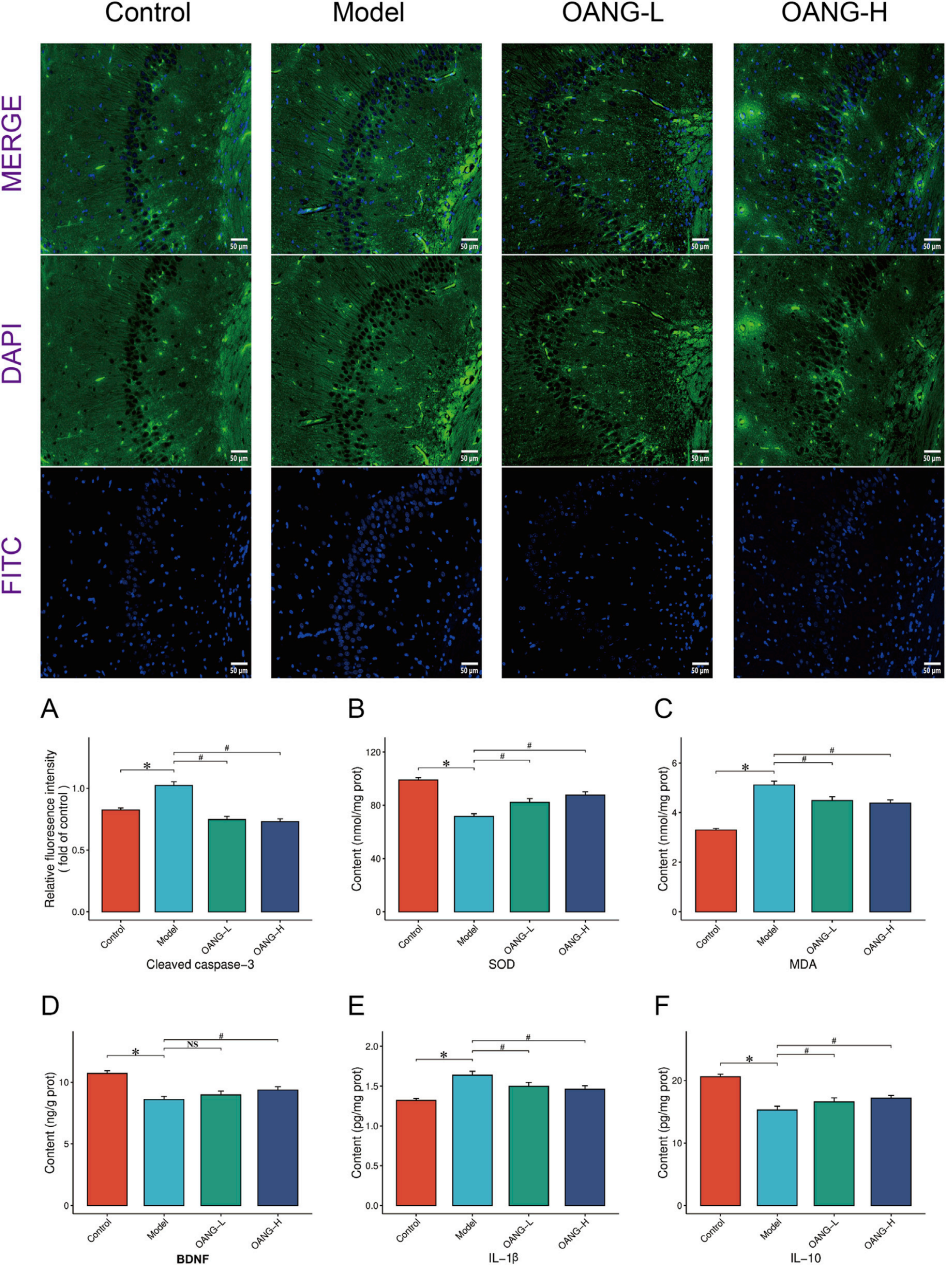
Results of hippocampal tissue examination. **(A)** Impact of OA on cleaved caspase-3 fluorescence intensity in the hippocampal regions of rats. **(B–F)** Results of various inflammatory factors in hippocampal.

Immunofluorescence staining indicated a regular fluorescence distribution pattern in the control group, which was disrupted in the model group (Figure 7). Both OANG-L and OANG-H ameliorated this abnormality, with OANG-H producing a distribution closer to that of the control group, indicating a dose-dependent effect.

Specifically, the model group showed significantly elevated levels of cleaved caspase-3 and MDA, and SOD, BDNF, and IL-10 (p < 0.05). OANG treatment significantly reduced cleaved caspase-3 and MDA, and increased SOD and IL-10 levels (p < 0.05). BDNF showed an increasing trend but did not reach statistical significance. The effects of OANG-L and OANG-H on these markers were comparable, with no significant inter-dose differences, indicating that OANG alleviates hippocampal apoptosis, oxidative stress, and inflammation at both dose levels.

## Discussion

This study systematically evaluated its therapeutic effects in a rat model of KOA. Behavioral, imaging, and histological analyses consistently demonstrated that OANG significantly alleviated pain and depression-like behaviors, reduced cartilage wear and subchondral bone sclerosis, and modulated inflammatory factors and oxidative stress indicators in the synovial fluid. Western blot and immunohistochemistry results further revealed that OANG exerts chondroprotective effects by upregulating type II collagen, suppressing MMP13, and modulating key proteins such as GSK-3β and SOX9. Network pharmacology and molecular docking suggested that OA might interact with multiple targets, including PPARG, MAPK3 and PTGS2, thereby participating in the regulation of inflammatory, metabolic, and neural signaling pathways (Wu et al., 2025a). Notably, OANG also mitigated oxidative stress and inflammatory responses in hippocampal tissue, indicating a systemic protective effect (Balde and Nazeer, 2025).

In recent years, the research perspective on KOA has shifted from a focus solely on mechanical wear to a view of it as a systemic disease, involving chronic low-grade inflammation, metabolic disturbances, and aberrant cell signaling (Peng et al., 2024). This study not only validated the therapeutic potential of OA but also overcame its poor solubility and short half-life through the nanogel delivery system, achieving sustained release and retention within the joint cavity. Mechanistically, OANG significantly inhibited pro-inflammatory cytokines such as TNF-α and IL-1β, likely related to its modulation of core inflammatory pathways like NF-κB and MAPK (Li et al., 2025a; Li et al., 2025b; Wang et al., 2025c). This subsequently led to the downregulation of downstream inflammatory mediators, including COX-2, iNOS, and related products (Wang et al., 2025d). Concurrently, OANG effectively enhanced the activity of antioxidant enzymes such as SOD and GSH-Px, suggesting the potential activation of the Nuclear Factor Erythroid 2–Related Factor 2/Antioxidant Response Element (Nrf2/ARE) pathway (Jiang et al., 2025). Changes in GSK-3β expression and the restoration of β-catenin nuclear localization indicated involvement of the Wnt/β-catenin pathway (Tong et al., 2025a). OANG may promote chondrocyte anabolism by appropriately inhibiting GSK-3β and stabilizing β-catenin, while avoiding pathway overactivation that leads to hypertrophic differentiation (Huang et al., 2025).

Network pharmacology provided a systematic perspective for understanding the multi-target action of OA. The 56 identified overlapping targets and their enriched pathways—such as arachidonic acid metabolism, estrogen signaling pathway, and cAMP signaling pathway—suggest that OA’s effects extend beyond mere anti-inflammation (Bao et al., 2020). For instance, the key target PPARG links OA to lipid metabolism and insulin sensitivity, hinting at its ability to enhance anti-inflammatory capacity by modulating the energy metabolic state of chondrocytes (Shi et al., 2021a). PTGS2 (i.e., COX-2) and MAPK3 (ERK1) directly correspond to OA’s role in inhibiting prostaglandin synthesis and regulating cell proliferation/apoptosis signals (Gao et al., 2025). The enrichment of ESR1 further suggests OA’s potential for sex-dependent modulation of bone and cartilage metabolism (Zhang et al., 2025b). Of particular interest is the enrichment of pathways like “neuroactive ligand-receptor interaction” and “serotonergic synapse,” which provided predictive clues for the central effects observed in the hippocampus (Tong et al., 2025b). This implies that OA may act as a “metabolism-neuro-immunity” modulator, achieving synergistic treatment of joints and the central nervous system through common signaling nodes (Chen et al., 2026).

In the hippocampus of KOA model rats, we observed increased levels of the apoptosis marker cleaved caspase-3, alongside decreased levels of BDNF and the anti-inflammatory cytokine IL-10, confirming central nervous system abnormalities associated with KOA and supporting the existence of a “brain-bone axis” (Li et al., 2025c). OANG reversed these changes, demonstrating its systemic protective effects. Mechanistically, persistent peripheral joint inflammation may lead to systemic increases in pro-inflammatory cytokines like IL-1β and TNF-α, which could affect the hippocampus via the blood-brain barrier or vagal afferent pathways, triggering microglial activation, exacerbated oxidative stress, and reduced secretion of neurotrophic factors (Jiang et al., 2026). The decrease in BDNF not only affects neuronal survival and plasticity but may also exacerbate pain and depression-like behaviors through descending modulation, forming a vicious cycle (Zhong et al., 2025). The cAMP signaling pathway, predicted by network pharmacology, could be a key hub connecting the joint and the brain, as it participates in both chondrocyte metabolism regulation and hippocampal synaptic plasticity and BDNF expression (Lipina et al., 2024). OANG might exert simultaneous protective effects at both the joint and hippocampal levels by co-upregulating this pathway, offering a new perspective for understanding KOA comorbidities (such as depression) and their treatment.

In summary, the mechanisms of OANG in treating KOA primarily involve three interrelated aspects. Regarding inflammatory regulation, it inhibits pathways like NF-κB and MAPK, downregulates COX-2/PGE2, iNOS/NO, and various interleukins, effectively reducing synovial and cartilage inflammation (Wu et al., 2025b). Concerning metabolic and oxidative stress balance, it likely activates the PPARγ and Nrf2/ARE pathways, modulating lipid metabolism and antioxidant gene expression to enhance cellular stress tolerance (Luo et al., 2025). Pertaining to cartilage metabolic homeostasis and neuro-cartilage interaction, it influences the balance between cartilage anabolism and catabolism by regulating the GSK-3β/β-catenin balance, and mediates central-peripheral crosstalk through neuro-related signals like cAMP and serotonin, thereby alleviating pain and depression-like manifestations (Cheng et al., 2023).

This study has several limitations. Methodologically, the papain-induced acute chemical injury model differs from the chronic natural progression of human KOA. Theoretically, only a portion of the numerous targets predicted by network pharmacology have been preliminarily verified, lacking functional experiments such as gene knockout or inhibitor interference to confirm the specific contribution of each target (Zhu et al., 2024). Experimentally, the detection of key nodal proteins (e.g., phosphorylation levels, nuclear/cytoplasmic distribution) in pathways like Wnt/β-catenin and Nrf2 remains insufficient (Wei et al., 2025). The exploration of the “brain-bone axis” remains correlative, lacking causal evidence such as central drug administration (Chen et al., 2022). Future research should focus on in-depth validation of specific pathways and comprehensively elucidate the systemic mechanism of OANG by integrating multi-omics technologies.

## Conclusion

This study demonstrates that OANG effectively delays the progression of KOA through multiple mechanisms, including local joint anti-inflammatory and antioxidant effects, protection of the cartilage matrix, and systemic regulation of the “brain-bone axis.” Integrating experimental validation and network pharmacology analysis, OA’s therapeutic actions involve several key pathways related to inflammation, metabolism, and neural signaling, reflecting a multi-target synergistic therapeutic advantage. As a locally injectable formulation, OANG holds translational potential, though future studies in chronic disease models are needed to further clarify its molecular pathways and long-term safety profile.

## Data Availability

The original contributions presented in the study are included in the article/supplementary material, further inquiries can be directed to the corresponding authors.

## Glossary

5-HT: 5-hydroxytryptamine
ARE: Antioxidant response element
BCA: Bicinchoninic acid
BDNF: Brain-derived neurotrophic factor
BMD: Bone mineral density
BV: Bone volume
CAT: Catalase
COMP: Cartilage Oligomeric Matrix Protein
CTX-II: Cross-linked C-telopeptide of type II collagen
CX: Celecoxib
ECL: Enhanced chemiluminescence
ELISA: Enzyme-Linked Immunosorbent Assay
ESR1: Estrogen receptor 1
GAPDH: Glyceraldehyde-3-phosphate dehydrogenase
GO: Gene Ontology
GSK-3β: Glycogen synthase kinase-3 beta
HE: Hematoxylin and eosin
IHC: Immunohistochemistry
IL-1β: Interleukin-1β
IL-6: Interleukin-6
IL-10: Interleukin-10
IL-17: Interleukin-17
IL-18: Interleukin-18
iNOS: Inducible nitric oxide synthase
KEGG: Kyoto Encyclopedia of Genes and Genomes
KOA: Knee osteoarthritis
MAPK3: Mitogen-activated protein kinase 3
MAPKs: Mitogen-activated protein kinases
MDA: Malondialdehyde
MMP13: Matrix metallopeptidase 13
NF-κB: Nuclear factor-kappa B
NSAIDs: Nonsteroidal anti-inflammatory drugs
OA: Oleanolic acid
OANG: Oleanolic acid-loaded liquid crystalline nanogel
OANG-H: OANG high dose
OANG-L: OANG low dose
PAGE: Polyacrylamide gel electrophoresis
PBS: Phosphate buffered saline
PEG: Polyethylene glycol
PMSF: Phenyl-methyl-sulfonyl fluoride
PPARA: Peroxisome proliferator-activated receptor alpha
PPARG: Peroxisome proliferator-activated receptor gamma
PPI: Protein-protein interaction
PTGS2: Prostaglandin-endoperoxide synthase 2
PVDF: Polyvinylidene fluoride
RIPA: Radioimmunoprecipitation assay
SDS: Sodium dodecyl sulfate
SOD: Superoxide dismutase
TEM: Transmission Electron Microscope
TGF-β: Transforming growth factor beta
TNF-α: Tumor necrosis factor-α
WB: Western blot
YAP: Yes-associated protein
